# Inovação tecnológica em artroplastia total do joelho: Navegação, robótica e customização

**DOI:** 10.1055/s-0045-1810045

**Published:** 2025-11-04

**Authors:** Marcus Vinicius Malheiros Luzo, Marcio de Castro Ferreira, Alexandre Barbieri Mestriner, Idemar Monteiro de Palma, Carlos Eduardo da Silveira Franciozi, Marcelo Seiji Kubota

**Affiliations:** 1 Grupo de Cirurgia do Joelho, Departamento de Ortopedia e Traumatologia, Escola Paulista de Medicina, Universidade Federal de São Paulo, São Paulo, SP, Brasil; 2 Grupo de Cirurgia do Joelho, Hospital Maradei, Belém, PA, Brasil

**Keywords:** artroplastia do joelho, cirurgia robótica, osteoartrite do joelho, próteses e implantes, arthroplasty, replacement, knee, osteoarthritis, knee, prostheses and implants, robot surgery

## Abstract

A artroplastia total de joelho (ATJ) é o procedimento padrão-ouro para o tratamento das patologias degenerativas articulares do joelho sem melhora com o tratamento conservador, e resulta em desfechos clínicos e funcionais excelentes. No entanto, uma parcela pequena dos pacientes relata alguma insatisfação com seus joelhos após as cirurgias. Neste contexto, nos últimos anos, o desenvolvimento tecnológico associado a este tratamento tem passado por relevante introdução de novos conceitos biomecânicos e incorporações de tecnologias que buscam o aprimoramento das técnicas cirúrgicas associado à evolução de desenhos de implantes que otimizem as características individuais dos pacientes para permitir melhor customização cirúrgica. O objetivo deste artigo é apresentar conceitos, vantagens e limitações associadas ao desenvolvimento dos implantes customizados, além das técnicas cirúrgicas de ATJ associada à automação (robótica) e à navegação assistida por computação. Ainda que exista ampla literatura médica que demonstra resultados promissores com as novas tecnologias nas cirurgias de ATJ, são necessários estudos com maior seguimento e metodologias adequadas a fim de contribuir com a demonstração de vantagens nos desfechos cirúrgicos nas artroplastias de joelho.

## Abstract (en)

Total knee arthroplasty (TKA) is the gold standard to treat degenerative knee joint conditions that do not improve with conservative treatment, and it yields excellent clinical and functional outcomes. However, a small proportion of patients report some dissatisfaction with their knees after surgery. Recently, TKA underwent significant technological development with the introduction of new biomechanical concepts and the incorporation of techniques to improve surgical procedures. Moreover, the evolution in implant designs optimized for the individual characteristics of the patients enables better surgical customization. The current article presents the concepts, advantages, and limitations to the development of customized implants, as well as TKA surgical techniques involving automation (robotics) and computer-assisted navigation. Although the medical literature showing promising outcomes with new TKA technologies is extensive, studies with longer follow-up and appropriate methodologies are required to contribute to the demonstration of advantages in knee arthroplasty outcomes.

## Introdução

A artroplastia total de joelho (ATJ) é a cirurgia considerada padrão-ouro para o estágio final da gonartrose, e, embora os desfechos clínicos deste procedimento sejam amplamente favoráveis, estima-se que aproximadamente 10% dos pacientes se mantêm insatisfeitos com a cirurgia. 1 2

Diante da crescente demanda dessa cirurgia e, levando-se em consideração o fato de que elas são realizadas cada vez mais em pacientes adultos jovens, 3 é notória a necessidade e a busca de resultados cirúrgicos de maior qualidade visando a longevidade dos implantes, a percepção mais fisiológica da articulação, a melhor funcionalidade do membro inferior, e menores traumas cirúrgicos. 4

Nesse contexto, novos avanços tecnológicos, tais como instrumentação e guias específicos, e a cirurgia assistida por computador (navegada) e/ou automação (robótica) buscam melhor customização e levar em conta a fisiologia e o equilíbrio dos ligamentos de cada paciente a fim de melhorar o planejamento cirúrgico tridimensionalmente, para aumentar a precisão do alinhamento e do posicionamento do implante. 4

Esses conceitos estão sendo apresentados e introduzidos no mercado assistencial das artroplastias com o propósito de melhorar a compreensão mecânica do membro inferior, o mapeamento articular e funcional do joelho, assim como a precisão e a confiabilidade cirúrgicas para aumentar a satisfação dos pacientes. No entanto, como a incorporação de tecnologias novas geralmente está associada a custos maiores, é fundamental que os cirurgiões compreendam as inovações para as ATJs para que desenvolvam senso crítico em relação às suas práticas e visualizem como usar adequadamente essas novas ferramentas para melhorar os resultados cirúrgicos. 5 6

O objetivo deste artigo é descrever as novas tecnologias em ATJ associadas ao uso da navegação, da robótica e dos implantes customizados, e discutir seus conceitos atuais, vantagens e limitações, para auxiliar o leitor a identificar como elas podem contribuir para o desenvolvimento técnico e melhorar os desfechos das artroplastias do joelho.

## Implantes Customizados

Os primeiros dispositivos para artroplastia do joelho foram introduzidos na década de 1970; desde então, foram submetidos a diversas alterações, que incluem técnica cirúrgica e desenho dos implantes. 7 Um implante é produzido com base nos parâmetros dimensionais anatômicos de um grupo populacional definido, o que pode levar a inadequações de ajuste entre os componentes protéticos e a anatomia articular em outras populações. Estudos realizados em populações não caucasianas, como indianos, chineses, malaios e coreanos, por exemplo, 8 9 10 11 12 demonstraram relações antropométricas diversas e diferentes das apresentadas por caucasianos. Na comparação destas populações, foram identificadas discrepâncias significativas com os modelos de próteses comerciais, o que pode ter efeito direto sobre os resultados da artroplastia. 8 9 10 11 12 A anatomia pode variar com o gênero, a etnia, o biotipo e as alterações fenotípicas adquiridas.

O desenvolvimento tecnológico promove terapias ortopédicas mais “personalizadas” e centradas nas condições próprias de cada indivíduo. Embora atualmente se trate de uma tendência, a individualização terapêutica remonta a Hipócrates, em seus dizeres: “é mais importante saber que tipo de pessoa tem uma doença do que saber que tipo de doença uma pessoa tem”. 13

A medicina individualizada está cada vez mais presente no campo das artroplastias, na busca por melhores desfechos. A ATJ personalizada é estimulada por quatro grandes pilares: 1) eixo mecânico mais adaptado à biomecânica individual; 14 15 2) variações anatômicas que não se enquadram em formatos de implantes predefinidos devido à variabilidade fenotípica articular; 3) evolução tecnológica que permite maior e melhor conhecimento e avaliação biomecânica intraoperatória; e 4) a pressão comercial dos fabricantes para massificar a utilização de seus produtos novos. 16

Nesse contexto, os componentes customizados foram desenvolvidos para um revestimento fisiológico que respeite a geometria e os limites ósseos, para evitar hiper ou hipodimensionamento anatômico e reduzir as chances de impactos e atritos em partes moles ou falta de cobertura articular adequada, associado a uma cinemática mais fisiológica. No entanto, ainda que se defenda o restabelecimento anatomofisiológico articular individualizado, é preciso refletir que, por vezes, a biomecânica natural de determinado paciente pode ser fator causal, ou de risco, para a dor e a disfunção articular.

Os implantes customizados permitem definições próprias do sulco troclear de maneira personalizada, o que favorece a cinemática patelofemoral independentemente dos formatos condilares femorais, que também podem ser fabricados com raios de curvaturas individuais. Essa particularização que potencializa benefício cinemático ao implante pode ainda contribuir com menor necessidade de compensação para modificar a rotação femoral para o equilíbrio do espaço ( *gap* ) de flexão. Obviamente, esta condição seria dependente da ausência de assimetrias tensionais ligamentares, que exigiriam liberação de partes moles a fim de se restabelecer o equilíbrio do *gap* . 17 18 19 O restabelecimento fisiológico e anatômico da curvatura condilar posterior aumenta os espaços de flexão e extensão. É preciso destacar a influência do raio de curvatura condilar femoral, porque sua relação com a cápsula posterior também interfere no dimensionamento do *gap* de extensão, condição que o cirurgião deve ter em mente ao dimensionar a projeção posterior ( *off set)* no planejamento de artroplastia em pacientes com joelhos em flexo ou hiperextensão. 20 21 Na busca por uma artroplastia fisiológica, em que o paciente não perceba seu joelho “artificial” nas rotinas diárias, desenvolveu-se um questionário para a mensuração do desfecho. 22

A ATJ personalizada deve, conceitualmente, manter ou procurar restaurar: 1) a percepção de uma articulação normal; 2) a biomecânica “funcional” (quando a anatomia nativa não é considerada patológica), a saber: a) os eixos cinemáticos nativos; b) a orientação nativa da linha articular; c) a tensão nativa de partes moles; e d) a cinemática e cinética articulares nativas durante atividades diárias; 3) a transferência adequada de estresse mecânico entre as interfaces; 4) o arco de movimento articular nativo; 5) a macro e microestabilidades articulares; 6) a resistência do polietileno ao desgaste e à fixação sólida do implante, o que contribui para sua sobrevida sem restrição às atividades; e 7) proporcionar uma recuperação rápida e sem complicações. 2

Diante dos novos desafios conceituais que os implantes customizados trazem com relação aos princípios técnicos clássicos da cirurgia de ATJ, binários entre a técnica da ressecção mensurada ou técnica do equilíbrio dos espaços ( *gap balancing* ), 23 Lee et al. 24 propuseram um conceito novo, dissociado dos princípios dos cortes perpendiculares aos eixos mecânicos femoral e tibial, o qual foi denominado de *alinhamento cinemático* , e visa obter perfil articular mais próximo da articulação nativa ao recriar os três eixos cinemáticos do joelho, que são paralelos e perpendiculares às linhas nativas da articulação. Posteriormente, outros conceitos foram introduzidos, como os alinhamentos cinemático invertido, cinemático irrestrito, funcional, e cinemático modificado. 24 25 O que une todas essas novas definições técnicas é a manipulação das tensões e linhas articulares mais “fisiológicas” e potenciais compensações, nos cortes ósseos, de pequenas assimetrias das tensões ligamentares, ou seja: os conceitos outrora estabelecidos por Insall et al. 26 hoje são questionados, se devemos restaurar toda a anatomia e frouxidão ligamentar nativas. As inovações tecnológicas, como a cirurgia assistida por computação (CAC), e as automações favorecerem novas percepções e entendimentos que conduziram a este momento de desenvolvimento conceitual para uma abordagem específica do paciente e são promissoras para que a evolução e desfechos das ATJ sejam otimizadas. É preciso ressaltar que o alinhamento anatômico foi um conceito concebido nos anos 1980 por Krackow, Krena e Hungerford para ser utilizado com a prótese preservando o ligamento cruzado posterior, que acabou no esquecimento, muito por conta de desgaste precoce do tipo de polietileno polido utilizado à época. Com o advento de materiais novos e melhores associado às incorporações de tecnológica assistida por navegação e robótica, a técnica de alinhamento anatômico vem sendo mais bem compreendida e utilizada. 25

É preciso mencionar que o conceito de ATJ personalizada não é universalmente aplicável. A doença degenerativa artrítica pode, em muitos pacientes, levar a articulação e o membro a desenvolver deformidades e variações anatômicas e biomecânicas, tais como encurtamento e alongamentos de partes moles e deformidades extra e intra-articulares que dificultam ou inviabilizam a identificação da cinemática fisiológica a ser restabelecida. Além disso, é preciso senso crítico para analisar se a condição anatomomecânica e fisiológica do paciente pode interferir negativamente na longevidade dos implantes. 27 Sendo assim, Vendittoli et al. 2 afirmaram que a ATJ personalizada não significa reproduzir a anatomia do paciente rotineiramente, mas oferecer uma solução cirúrgica de melhor qualidade para abordar a doença com uma tecnologia customizada de implante.

Diante de todas as considerações e conceitos teóricos vantajosos apresentados sobre os implantes customizados, ainda faltam maiores evidências clínicas para demonstrar superioridade dos desfechos com esses tipos de implantes. Müller et al. 28 e Saeed et al. 29 demostraram que as ATJs personalizadas não proporcionaram benefícios superiores aos dos implantes convencionais, e eles observaram maior taxa de revisão precoce entre as customizadas. Já Moret et al. 30 não encontraram diferenças significativas quanto ao desfecho clínico entre os implantes customizados e convencionais. Entretanto, os implantes personalizados se mostraram mais alinhados ao eixo neutro do membro e com melhores ajuste e posicionamento dos implantes.

É preciso ressaltar a necessidade de melhores metodologias de pesquisa e desenhos de estudo nas comparações prospectivas entre implantes customizados e convencionais, a fim de clarificar a compreensão dos desfechos da cirurgia personalizada e das técnicas clássicas.

## Artroplastia Assistida por Computação (Navegada) e Automação (Robótica)

No final dos anos 1970 e início dos anos 1980, os conceitos cirúrgicos e biomecânicos sobre o alinhamento resultante do membro e o equilíbrio ligamentar pós-artroplastia do joelho foram apresentados com maior solidez científica, o que favoreceu o desenvolvimento de implantes melhores e otimizou o desempenho cirúrgico. 26 No começo dos anos 1990, iniciou-se o desenvolvimento da técnica cirúrgica para a ATJ assistida por computação (AC). O princípio dessa tecnologia se baseava na identificação de referências anatômicas intraoperatórias, como os centros da cabeça femoral, do joelho e do tornozelo, para proporcionar visões frontal e lateral em tempo real do eixo mecânico do membro inferior para um melhor controle intraoperatório do eixo do membro do paciente, sendo a primeira ATJ navegada realizada em 21 de janeiro de 1997. 31 Essa tecnologia tem como premissa facilitar a interpretação anatomomecânica do membro do paciente de modo a favorecer as decisões técnicas a serem adotadas pelo cirurgião para definir a melhor estratégia para a cinemática articular e o posicionamento dos implantes.

As ATJs assistidas por navegação foram evoluindo tecnologicamente e permitiram ao cirurgião identificar, no intraoperatório, muito mais do que o eixo resultante final do membro, uma vez que passaram a permitir a realização com precisão das angulações dos cortes femoral e tibial, o posicionamento rotacional dos componentes femoral e tibial, as dimensões dos espaços de extensão e flexão, e os dimensionamentos dos implantes. A introdução da tecnologia AC associada à ATJ permitiu ao cirurgião ampla compreensão, domínio articular intraoperatório e maior precisão do procedimento em relação ao planejamento, e esse conhecimento contribuiu para o desenvolvimento de novos conceitos mecânicos do alinhamento resultante dos membros. 25 Tais alinhamentos foram flexibilizando os conceitos referentes aos cortes femoral e tibial outrora perpendiculares ao eixo mecânico no alinhamento mecânico convencional. Essa flexibilização permite a estabilidade articular ao diminuir ou evitar liberações de partes moles, ou seja, ao permitir um ajuste de corte ósseo “fino” que compensa pequenos desequilíbrios ligamentares mediante a inclinação da linha articular em relação ao eixo mecânico, se aproximando da anatomia do joelho nativo. 14

Ao identificar a possibilidade de se aumentar a precisão cirúrgica da ATJ AC quanto aos cortes ósseos, a cirurgia ortopédica robótica, nos últimos anos, passou por relevante desenvolvimento, e conseguiu aumentar a qualidade e a praticidade do procedimento. 32

A ATJ robótica (ATJr) usa um programa de computador para converter os registros anatômicos do paciente em uma reconstrução tridimensional da articulação do joelho, o que permite que o cirurgião planeje e execute a cirurgia com precisão quanto aos cortes definindos. Portanto, a ATJr é uma técnica cirúrgica que combina navegação AC e auxílio da automação robótica para o posicionamento e/ou realizações dos cortes. 14 32 33 34 A ATJr pode ser dividida em dois subgrupos: a ATJr ativa (autônoma), em que a automação é completa e independe da atuação do cirurgião para a realização dos cortes femorais e tibial planejados; e o sistema robótico semiativo (semiautônomo), mais difundido globalmente e presente no Brasil neste momento, no qual o cirurgião mantém o controle geral sobre a ressecção óssea assistido pela precisão da automação por meio do posicionamento dos guias de cortes ósseos ou de um braço robótico que auxilia a controlar a força, a amplitude de alcance e a direção da lâmina de serra dentro dos limites programados. 34

Pode-se afirmar, de maneira resumida e sucinta, que a principal diferença conceitual entre uma ATJ AC e uma ATJr jaz no fato de a primeira ser um procedimento em que o cirurgião posiciona os guias e realiza os cortes assistido por monitoramento computacional. E a ATJr é uma ATJ AC com o auxílio da automação para o posicionamento dos guias de cortes e/ou a realização dos cortes ósseos. O principal benefício da ATJr é o corte ósseo preciso e reprodutível devido a uma interface robótica, independentemente do sistema usado. Além disso, ela permite a mensuração dos *gaps* de acordo com o planejamento dos cortes ósseos e o posicionamento do implante durante a cirurgia. 4 A precisão dos cortes ósseos conforme planejados é o principal benefício da ATJr, o que contribui para o posicionamento ideal dos implantes e o equilíbrio articular pela mensuração exata dos espaços de flexão e extensão.

Não existem indicações e contraindicações absolutas para a realização da ATJr ou da ATJ AC. No entanto, essas tecnologias se destacam e apresentam vantagens em relação a técnica convencional nos casos de pacientes que apresentam deformidades ósseas diafisárias e hastes intramedulares, condições que dificultam a realização da cirurgia pela técnica convencional.

Pacientes com deformidades articulares e falhas ósseas também podem ser favorecidos pelo uso da navegação, uma vez que essa tecnologia permite ao cirurgião visualizar as múltiplas possibilidades de de cortes e dimensionamentos dos implantes durante o planejamento e o intraoperatório.

Alguns sistemas de automação dependem de exame radiográfico pré-operatório para o programa de computador interpretar a reconstrução tridimensional articular que será conciliada com os pontos anatômicos identificados pelo cirurgião. No entanto, é possível, a depender do sistema AC robótico, que a cirurgia seja realizada sem a obrigatoriedade do mapeamento radiológico prévio, o que permite que todas as referências anatômicas e a identificação espacial do joelho e do membro inferior sejam capturadas integralmente durante o ato cirúrgico.

Destaca-se que a ATJ AC permite, com precisão, o controle crítico de muitos conceitos e elementos técnicos relevantes para o comportamento funcional do joelho, a fim de proporcionar previsibilidade mecânica e cinemática ao membro operado antes e após os cortes ósseos, a saber:

- a inclinação ( *slope* ) sagital posterior da tíbia na formação do espaço de flexão;
- o grau de rotação do componente femoral aplicável para a equalização do espaço de flexão entre os compartimentos femorotibiais medial e lateral, e potencial invasão da cortical femoral anterior ( *notching* );
- o dimensionamento e a proporcionalidade entre os espaços de extensão e flexão de acordo com os dimensionamentos do componente femoral e do polietileno tibial;
- o tamanho do componente femoral e sua relação com o dimensionamento do espaço de flexão;
- o posicionamento do implante femoral no plano sagital;
- a capacidade de intercambiar os implantes femoral e tibial;
- a angulação resultante do membro inferior em relação ao eixo mecânico pelos ajustes planejados;
- a resultante sagital do membro inferior em neutro, recurvo ou flexo;
- o dimensionamento dos cortes femorais distal e posterior e tibial proximal;
- a altura da interlinha articular;
- a equalização entre os espaços de flexão e extensão; e
- o dimensionamento do polietileno.

A literatura 35 36 37 38 apresenta resultados favoráveis e promissores quanto à capacidade de essa tecnologia proporcionar maior precisão dos componentes, proteção dos tecidos moles, maior satisfação do paciente, uma curva de aprendizado curta, desenho ergonômico ideal e menos fadiga do cirurgião e da equipe cirúrgica. Outros estudos demonstram resultados clínicos semelhantes aos das técnicas convencionais. Onggo et al., 32 em uma revisão sistemática que analisou mais de 6 mil pacientes, concluíram que tanto a ATJr quanto a cirurgia convencional são confiáveis, seguras, e resultam em bons desfechos, ainda que o alinhamento final do membro seja mais preciso nas robóticas. Em uma revisão, Mancino et al. 39 encontraram vantagem para o grupo de ATJr em termos de resultados funcionais iniciais e linhas de radiolucências em comparação ao grupo submetido ao procedimento convencional. Nenhuma diferença significativa entre os dois grupos foi relatada quanto à sobrevivência geral, à taxa de revisão e ao tempo cirúrgico. Em uma revisão sistemática com metanálise de 12 ensaios clínicos randomizados com mais de 2 mil pacientes analisados, Ruangsomboon et al. 40 concluíram que, apesar de a ATJr provavelmente resultar em maior precisão radiológica do que o procedimento convencional, este desfecho pode não ser clinicamente significativo.

Em uma revisão sistemática recente, Nogalo et al. 41 analisaram os relatos das complicações relacionadas às artroplastias robóticas. As principais complicações observadas foram fraturas metafisárias nos orifícios de fixação dos pinos dos refletores da navegação, com ∼ 1,4% delas ocorrendo próximo da décima segunda semana de pós-operatório. A infecção no local dos pinos de reflexões e em incisões acessórias também é descrita, com incidência de 0,47%. 42 É preciso ressaltar que a cirurgia robótica é segura. No entanto, complicações podem acontecer, sendo mais relacionadas à técnica cirúrgica aplicada pelo cirurgião do que por consequência direta da tecnologia empregada. 43

## Considerações Finais

Diante das considerações abordadas neste estudo referente aos propósitos da incorporação de novas tecnologias, assim como novos conceitos técnicos referentes à ATJ, percebe-se, no momento, que os avanços são promissores, ainda que haja a necessidade de estudos com metodologias de maior qualidade e com seguimentos mais longos, a fim de compreendermos as vantagens técnicas de médio e longo prazos quanto aos desfechos clínicos e, por consequência, permitir o desenvolvimento de técnicas e práticas melhores associadas a artroplastias de joelho.
